# Management of a pelvic impalement injury: a case report

**DOI:** 10.1093/jscr/rjaf462

**Published:** 2025-07-03

**Authors:** Wail Alqatta

**Affiliations:** Department of General and Visceral Surgery, Ibn Sina Hospital, Sanaa 12677, Yemen

**Keywords:** pelvic impalement, penetrating trauma, surgical management, diagnostic laparoscopy, soft tissue debridement, case report

## Abstract

Pelvic impalement injuries are uncommon but represent high-risk trauma requiring prompt, systematic evaluation and management. The proximity to major neurovascular structures and visceral organs necessitates a meticulous diagnostic approach. This report presents a 45-year-old male who sustained an impalement injury from a metallic rod traversing the left lower femoral region to the left chest wall. On presentation, the patient was hemodynamically stable. Computed tomography imaging excluded major vascular, abdominal, and thoracic injuries. Diagnostic laparoscopy confirmed the absence of intra-abdominal involvement. Surgical management involved controlled extraction of the foreign body, meticulous hemostasis, and extensive soft tissue debridement. The postoperative course was uneventful, with the patient regaining full limb function without neurological or vascular deficits. This case underscores the necessity of advanced imaging, structured operative planning, and multidisciplinary collaboration in the management of complex impalement injuries. Early, coordinated intervention is critical to optimize outcomes and minimize morbidity.

## Introduction

Impalement injury is a trauma caused by a blunt-tipped object, typically from falls, sexual activity, or slipping with external force [1]. It involves complex surgical challenges as it transfixes one or more body regions, combining blunt and penetrating trauma with significant tissue destruction and organ penetration [2]. Pelvic impalements are rare but severe, often occurring from falls, especially among construction workers [3], but also in sexual abuse and assaults [4].

These injuries carry high morbidity and mortality risks, particularly from pelvic bleeding due to vital blood vessel involvement, which can lead to hemorrhagic shock. Patient survival depends on factors such as pre-hospital care, blood loss, organ involvement, and effective blood replacement, along with management of late trauma complications [4]. There is no strict specific protocol to approach an impalement injury, due to the variability and complexity of each case [5].

This report describes a young male with a pelvic impalement injury from a metallic rod, which passed through subcutaneous and epifascial layers without damaging major vessels or organs. Computed tomography (CT) helped define the injury’s trajectory and rule out vascular or organ damage. Surgical management included foreign body extraction, hemostasis, and wound debridement. The patient recovered without neurological or functional deficits. This case emphasizes the importance of advanced imaging, a structured surgical approach, multidisciplinary collaboration, and careful postoperative monitoring for optimal outcomes in complex impalement injuries.

## Case information

**Figure 1:**
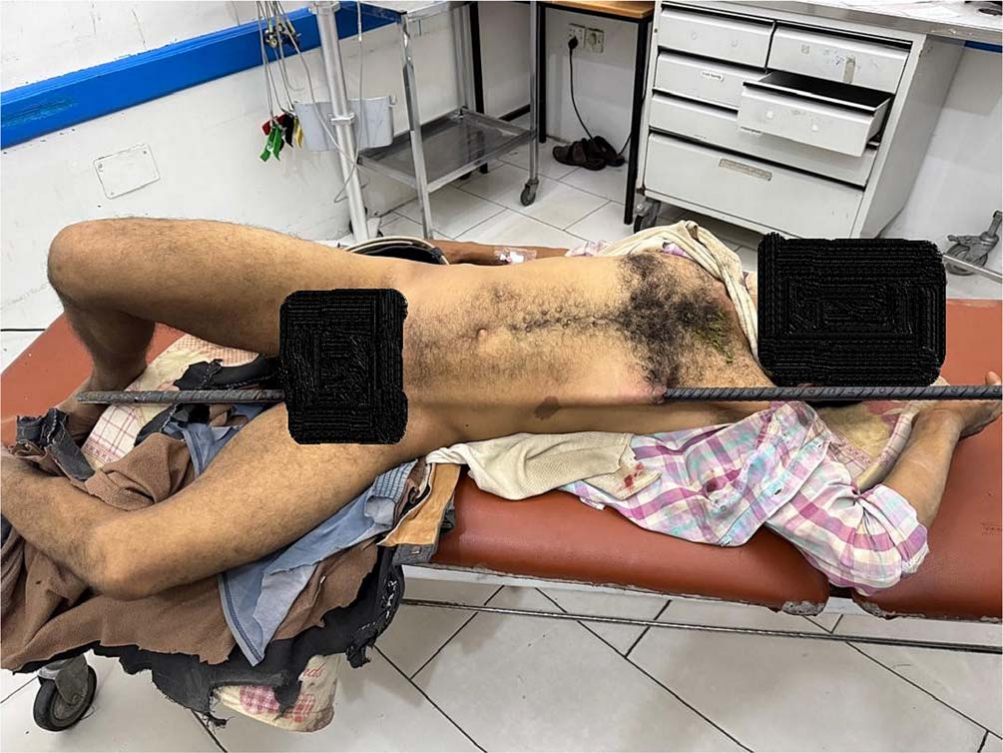
Clinical image of the patient upon arrival at the emergency department, showing a metallic rod impaling the pelvis and extending subcutaneously through the abdominothoracic region.

A 45-year-old construction worker sustained a severe pelvic impalement injury after falling onto a vertically positioned metal rod. He was left impaled and suspended, requiring the rod to be cut for his removal. Upon arrival at the emergency department, the patient was stable but in severe pain, for which he received analgesia (Fig. 1). He was prepared for surgery, with blood units arranged for potential blood loss. Laboratory results showed a hemoglobin level of 11.1 g/dl, with otherwise normal findings.

**Figure 2:**
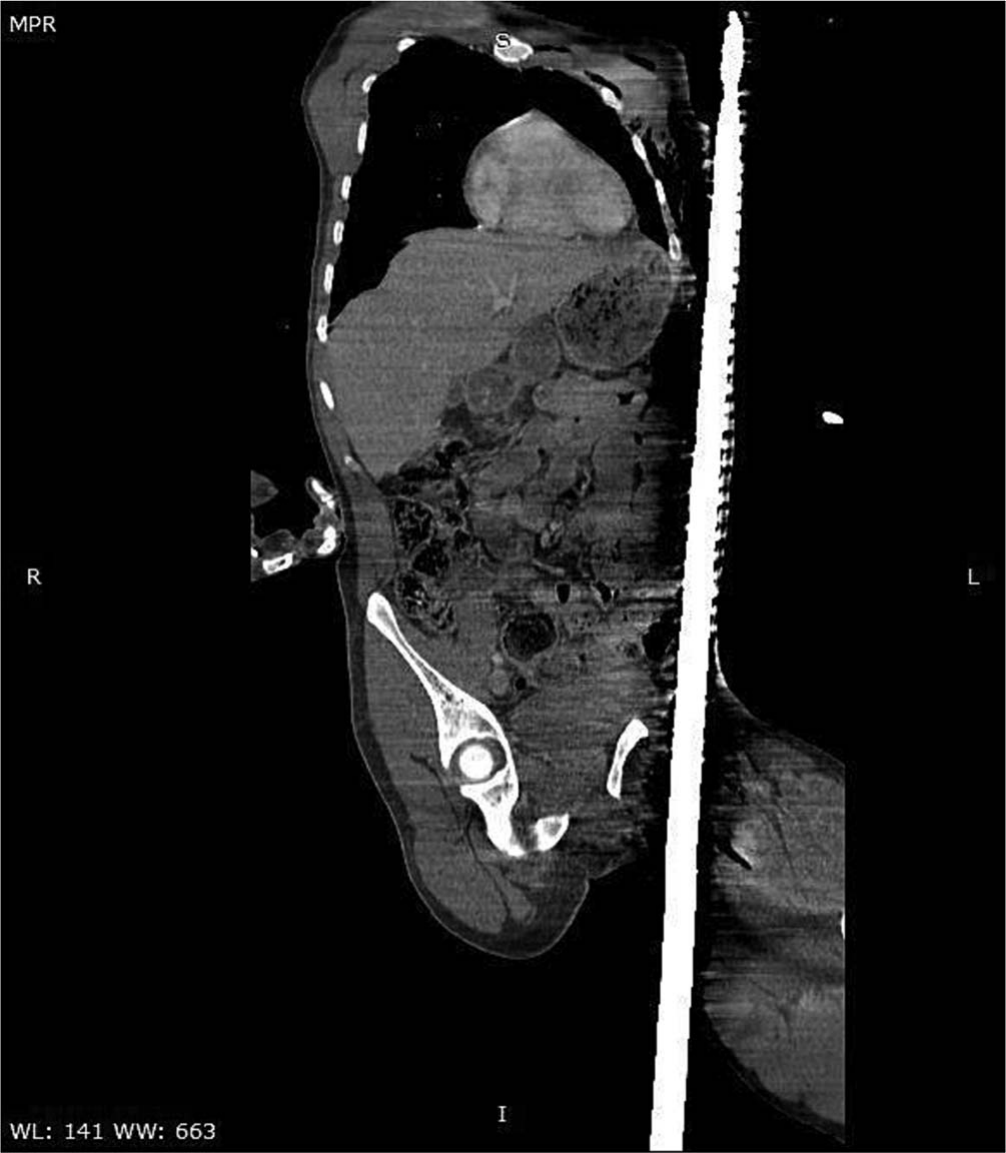
Sagittal CT image demonstrating the trajectory of the metallic rod traversing the body through the subcutaneous and epifascial planes.

**Figure 3:**
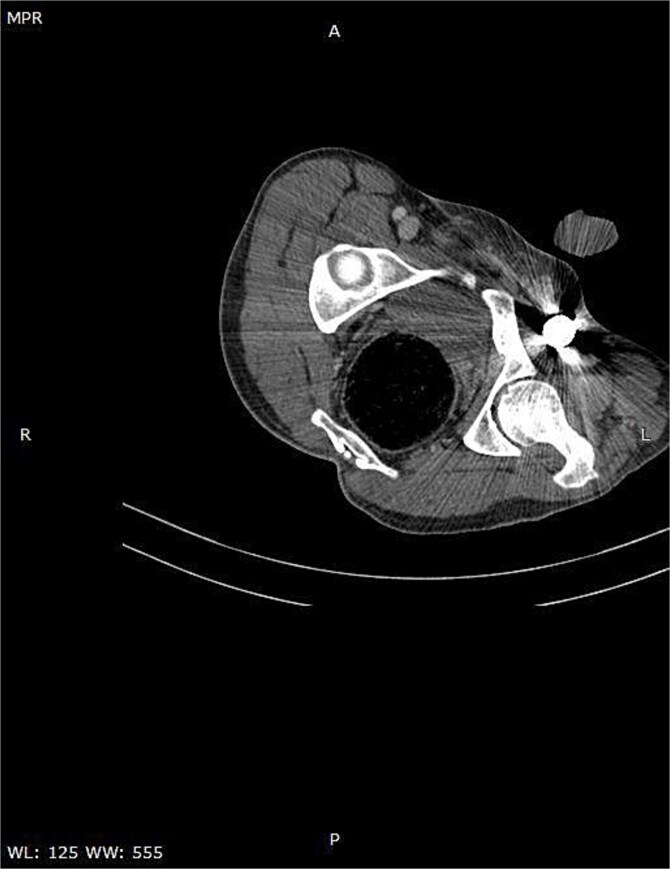
Axial CT image demonstrating the metallic rod positioned in close proximity to the femoral sheath without evidence of vascular injury.

**Figure 4:**
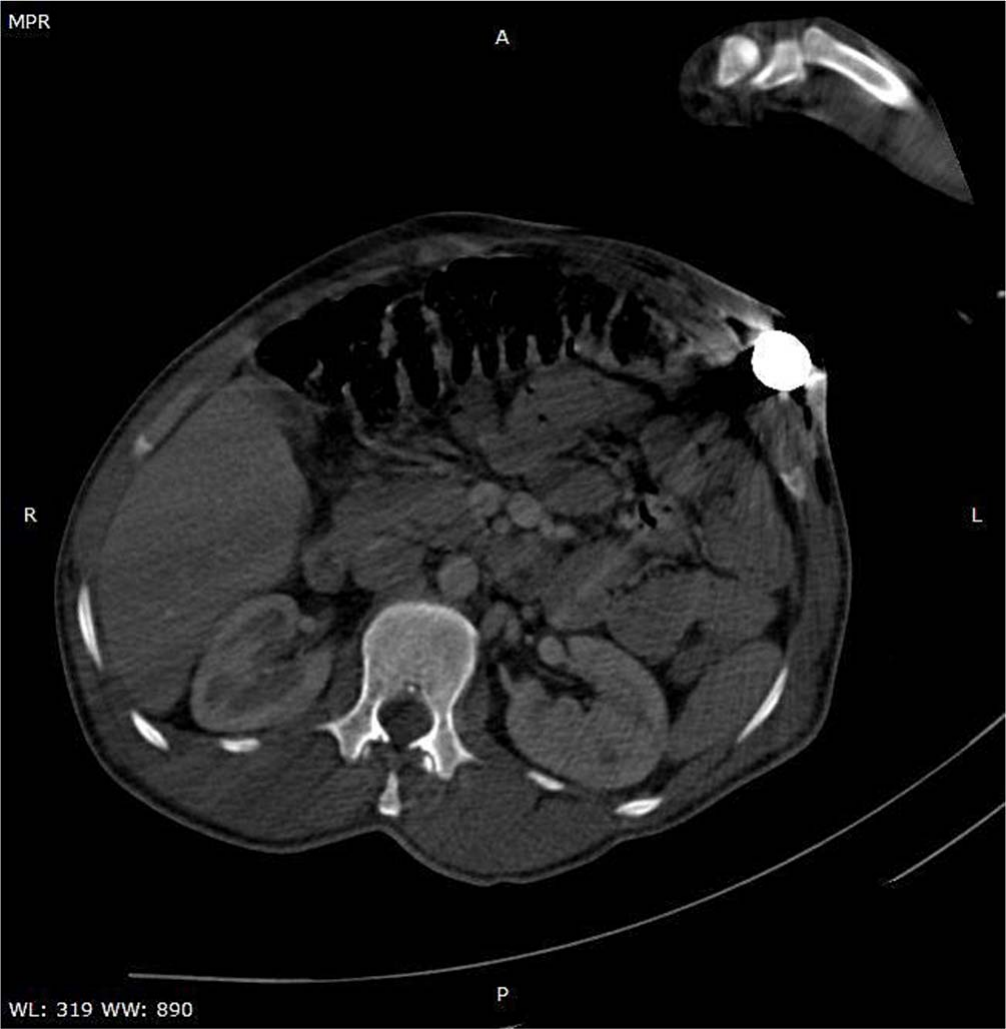
Axial CT image illustrating the entry site of the metallic rod through the abdominal wall.

A CT scan of the pelvis, abdomen, and chest revealed the metal rod’s trajectory, penetrating the medial aspect of the left proximal thigh and traversing the left inguinal region lateral to the femoral vessels and nerve, with no evidence of vascular injury or hematoma (Figs 2 and 3). The rod continued through the abdominal wall in the subcutaneous plane without entering the peritoneal cavity, and no free fluid was detected (Fig. 4). It then extended through the left chest wall, associated with subcutaneous emphysema, but no intrathoracic injury was evident.

**Figure 5:**
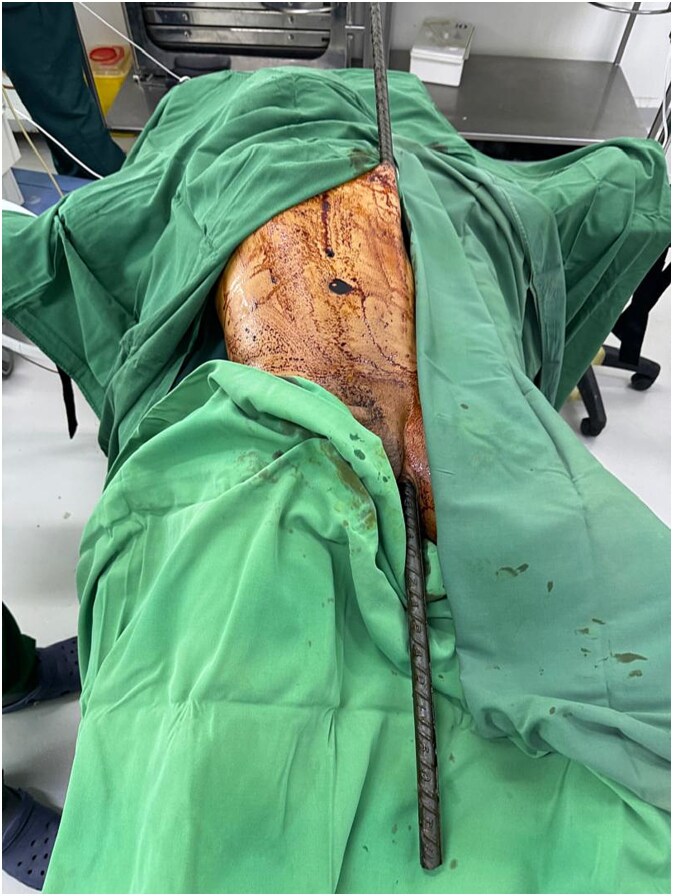
Intraoperative image showing the metallic rod traversing the patient’s body through the subcutaneous and epifascial planes.

**Figure 6:**
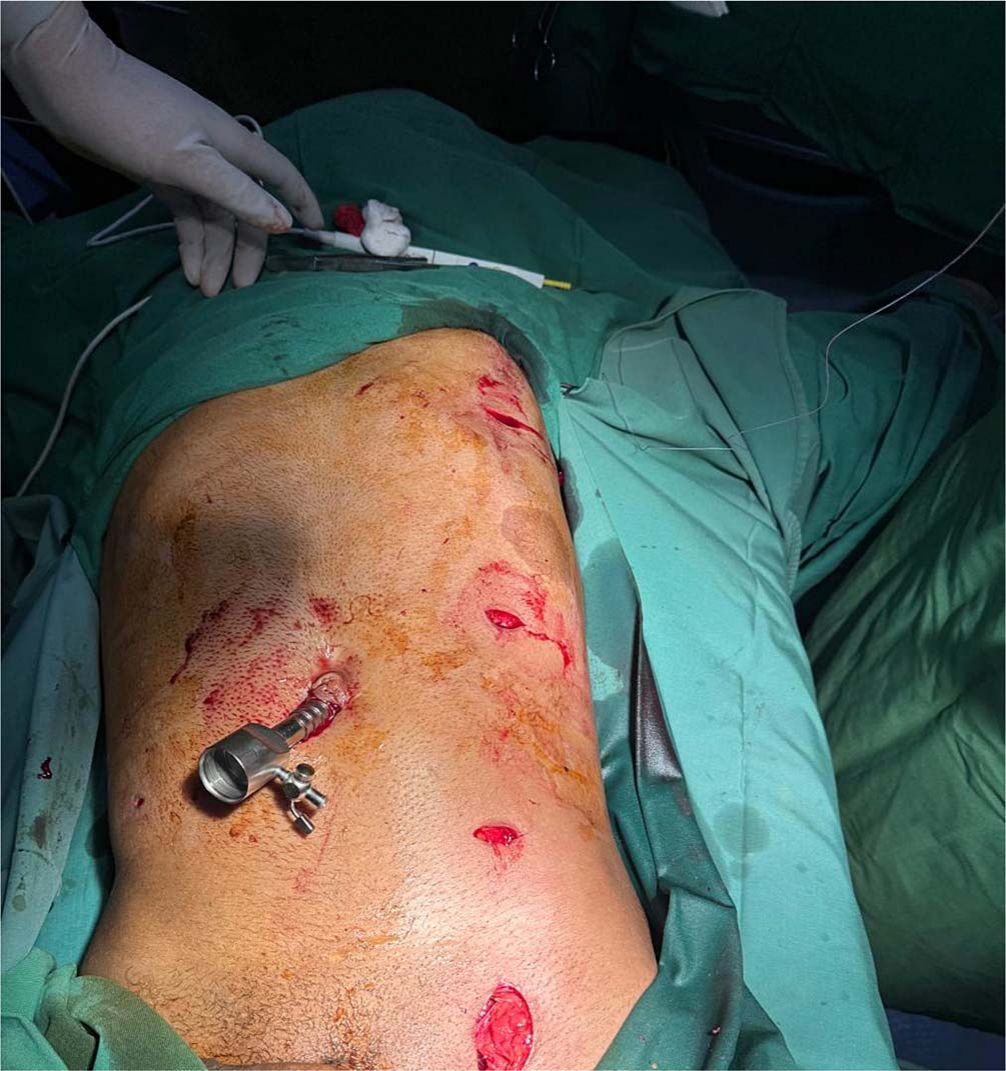
Intraoperative image demonstrating the completion of diagnostic laparoscopy and the successful extraction of the metallic foreign body.

**Figure 7:**
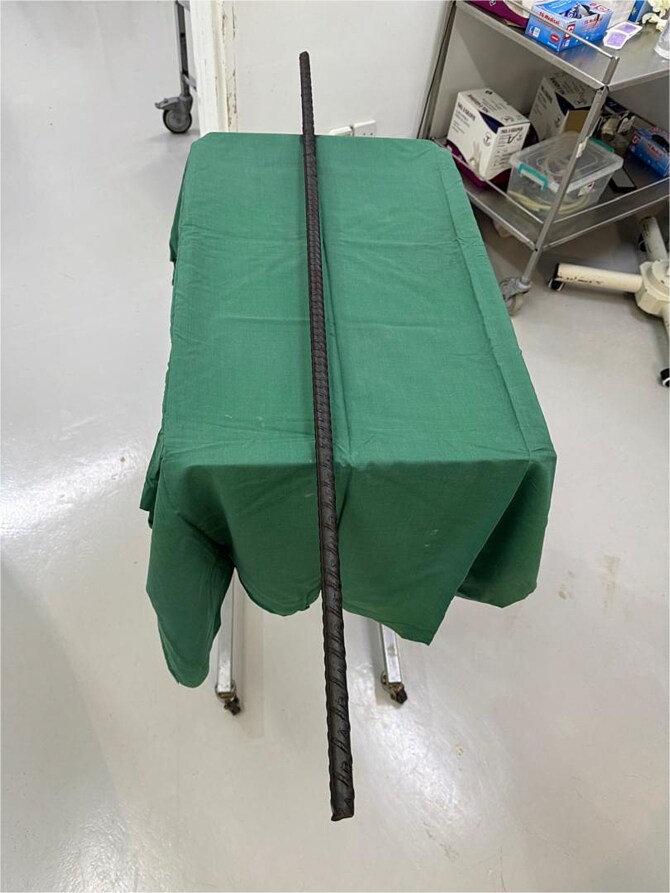
Intraoperative image showing the extracted metallic rod, measuring 1.5 m in length.

Given the injury’s complexity, surgical planning included a vascular surgeon on standby due to the rod’s proximity to major vessels (Fig. 5). Diagnostic laparoscopy confirmed the integrity of intra-abdominal organs, as the rod traversed and compressed the abdominal wall without penetrating the peritoneal cavity. Local exploration of the left femoral region showed the rod lateral to the femoral vessels and nerve with no acute bleeding or hematoma. Multiple small incisions allowed careful dissection to free the rod, enabling smooth extraction without significant bleeding (Figs 6 and 7).

Postoperatively, the patient experienced an uncomplicated recovery with no bleeding, infection, or neurological deficits. Early mobilization and enteral feeding were initiated on the first day, and the patient was discharged in stable condition on day four.

This case underscores the challenges of managing impalement trauma, highlighting the importance of meticulous surgical planning and a multidisciplinary approach to optimize outcomes.

## Discussion

Perineal impalement injuries involving the thoraco-abdomino-pelvic regions are rare and result from elongated, fixed objects penetrating the body, excluding firearm injuries. Historical use dates back to the Middle Ages, but modern cases often involve falls, motor vehicle collisions, sexual abuse, and iatrogenic injuries [6, 7]. These injuries are complicated by crush trauma, wound contamination, and infection [1].

Impalement injuries are classified into two types [8]:

- Type I: Result from the body impacting a stationary object (e.g. falls, vehicle collisions).
- Type II: Result from external manipulation, where the object is moving and the body is immobile (e.g. assaults, spears).

Perineal impalements are particularly severe, with high morbidity and mortality, as 25% of patients do not survive hospital admission [9]. Effective management requires a multidisciplinary approach, including pre-hospital care, immobilization, imaging studies, and proper trauma center transport [10]. Surgical intervention is common, though conservative treatment may apply to less severe cases [11, 12]. CT is the preferred imaging modality for perineal trauma [13, 14].

In our case, the impaled object spared major blood vessels and organs, leading to a favorable outcome. Despite this, pelvic impalement injuries should always be carefully managed due to the potential for unseen complications. Postoperative care, including ICU admission and antibiotic therapy, is critical, as complications such as fistulas, abscesses, and sepsis can occur in up to 80% of cases [15].

This case highlights the importance of a multidisciplinary approach to pelvic impalement injuries. Early CT and diagnostic laparoscopy excluded major vascular, visceral, and skeletal damage, enabling controlled foreign body extraction and minimizing morbidity. Postoperative monitoring and adherence to trauma and infection protocols ensured a positive outcome, emphasizing the value of timely, comprehensive assessment and management for optimal recovery.

## References

[1] Oya S, Miyata K, Yuasa N, et al. Impalement injury to the left buttock with massive bleeding: a case report. Nagoya J Med Sci 2013;75:147–52. 23544279 PMC4345709

[2] Horowitz MD, Dove DB, Eismont FJ, et al. Impalement injuries. J Trauma 1985;25:914–6. 10.1097/00005373-198509000-00017 4032518

[3] Sarica C, Yucetas SC, Ucler N, et al. Steel rod impalement injuries involving the spine: a case report and literature review. Ulus Travma Acil Cerrahi Derg 2019;25:417–23. 10.5505/tjtes.2018.83727 31297784

[4] Xie B, Leong CM, Joethy J, et al. Perineal impalement injury by steel bar: a near miss. Trauma Case Rep 2016;6:1–7. 10.1016/j.tcr.2016.09.004 29942851 PMC6013009

[5] Ugoletti L, Zizzo M, Castro Ruiz C, et al. Gluteal, abdominal, and thoracic multiple impalement injuries: a case report on management of a complex polytrauma. Medicine (Baltimore) 2019;98:e15824. 10.1097/MD.0000000000015824 31145320 PMC6709277

[6] Edwin F, Tettey M, Sereboe L, et al. Impalement injuries of the chest. Ghana Med J 2009;43:86–9. 10.4314/gmj.v43i2.55320 21326848 PMC3039238

[7] Bemelman M, Hammacher E. Rectal impalement by pirate ship: a case report. Injury Extra 2005;36:508–10. 10.1016/j.injury.2005.04.016

[8] Eachempati S . Impalement injuries. Duke Trauma Newsl 1998;2:6–7.

[9] Kudsk KA, McQueen MA, Voeller GR, et al. Management of complex perineal soft-tissue injuries. J Trauma 1990;30:1155–60. 10.1097/00005373-199009000-00012 2213949

[10] Malkomes P, Störmann P, El Youzouri H, et al. Characteristics and management of penetrating abdominal injuries in a German level I trauma center. Eur J Trauma Emerg Surg 2019;45:315–21. 10.1007/s00068-018-0911-1 29356844

[11] Lunevicius R, O’Sullivan A. Unusual management of thoracoabdominal impalement injury to the right hemiliver and diaphragm. Chin J Traumatol 2011;14:379–81. 24506923

[12] Schijns J, Plötz FB. A conservative approach in a child with haematuria after accidental rectal impalement trauma. Afr J Paediatr Surg 2015;12:191–2. 10.4103/0189-6725.170198 26612125 PMC4955428

[13] Demetriades D, Velmahos G. Technology-driven triage of abdominal trauma: the emerging era of nonoperative management. Annu Rev Med 2003;54:1–15. 10.1146/annurev.med.54.101601.152512 12471178

[14] Choe J, Wortman JR, Sodickson AD, et al. Imaging of acute conditions of the perineum. Radiographics 2018;38:1111–30. 10.1148/rg.2018170151 29906202

[15] Petrone P, Velandia WR, Dziaková J, et al. Treatment of complex perineal trauma: a review of the literature. Cir Esp 2016;94:313–22. 10.1016/j.ciresp.2015.11.010 26895924

